# Supplementary figures and images for: A multi-component, community-based strategy to facilitate COVID-19 vaccine uptake among Latinx populations: From theory to practice
Source: PLoS One. 2021 Sep 20;16(9):e0257111. doi: 10.1371/journal.pone.0257111 (PMC8452046; doi:10.1371/journal.pone.0257111)

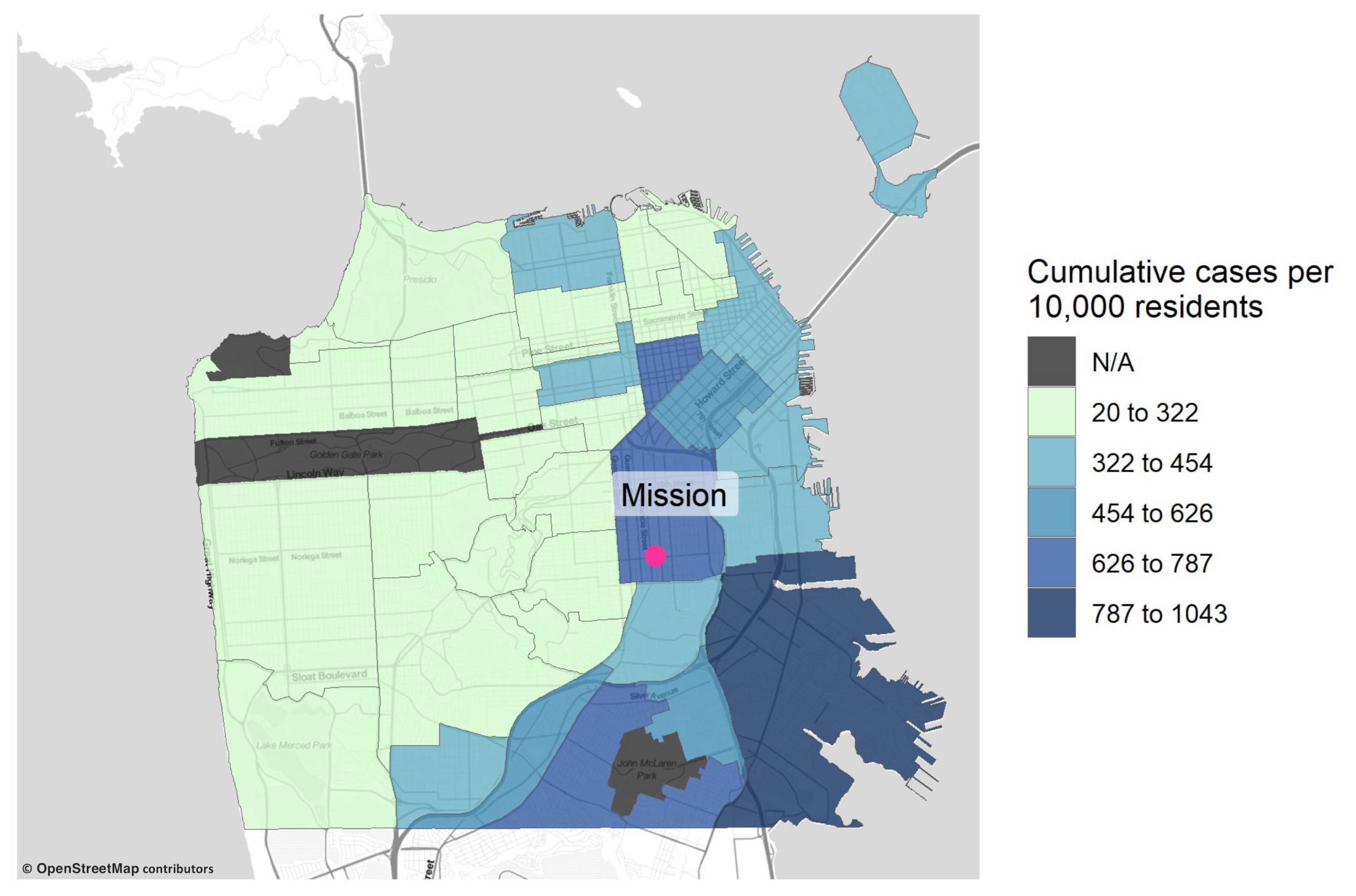

Supplement: S1 Fig — Darker blue shading indicates a higher cumulative prevalence of COVID-19 in a given neighborhood. The pink dot indicates the location of the Unidos en Salud neighborhood vaccination site. (TIF) [file pone.0257111.s001.tif]
