# Supplementary material for: A multi-component, community-based strategy to facilitate COVID-19 vaccine uptake among Latinx populations: From theory to practice
Source: PLoS One. 2021 Sep 20;16(9):e0257111. doi: 10.1371/journal.pone.0257111 (PMC8452046; doi:10.1371/journal.pone.0257111)
Supplement: S1 Table — (DOCX) [file pone.0257111.s002.docx]

**S1 Table. Characteristics of individuals who were registered for vaccination at the Unidos en Salud vaccination site between February 1 and May 19, 2021, according to whether they did or did not receive at least one vaccine dose at the Unidos en Salud vaccination site.**

|  | **Registered for vaccination at UeS site**  **(n=12,103)** | **Received at least one vaccine dose at UeS site**  **(n=11,098)** | **Did not receive at least one vaccine dose at UeS site**  **(n=1,005)** |
| --- | --- | --- | --- |
| **Median age, IQR** | 43 (32-56) | 43 (32-56) | 42 (30-54) |
| **Age Category** |  |  |  |
| 16-30 | 2796 (100%) | 2530 (90.5%) | 266 (9.5%) |
| 31-50 | 5064 (100%) | 4658 (92.0%) | 406 (8.0%) |
| 50-64 | 2850 (100%) | 2617 (91.8%) | 233 (8.2%) |
| 65 and older | 1393 (100%) | 1293 (92.8%) | 100 (7.2%) |
| **Sex** |  |  |  |
| Male | 5423 (100%) | 4926 (90.8%) | 497 (9.2%) |
| Female | 6469 (100%) | 5978 (92.4%) | 491 (7.6%) |
| Non-binary/other | 211 (100%) | 194 (91.9%) | 17 (8.1%) |
| **Ethnicity** |  |  |  |
| Latinx | 8458 (100%) | 7809 (92.3%) | 649 (7.7%) |
| White | 1716 (100%) | 1581 (92.1%) | 135 (7.9%) |
| Asian | 959 (100%) | 852 (88.8%) | 107 (11.3%) |
| Black | 313 (100%) | 269 (85.9%) | 44 (14.1%) |
| Other | 657 (100%) | 587 (89.3%) | 70 (10.7%) |

**Note:** proportions represent row percentages
