# Supplementary material for: A multi-component, community-based strategy to facilitate COVID-19 vaccine uptake among Latinx populations: From theory to practice
Source: PLoS One. 2021 Sep 20;16(9):e0257111. doi: 10.1371/journal.pone.0257111 (PMC8452046; doi:10.1371/journal.pone.0257111)
Supplement: S2 Table — (DOCX) [file pone.0257111.s003.docx]

**S2 Table. Geographic residence of clients receiving at least one vaccine dose at the Unidos en Salud neighborhood vaccination site between February 1 and May 19, 2021 according to zip code.**

|  | **Overall**  **(n=11,098)** | **Latinx**  **(n=7,809)** | **Not Latinx**  **(n=3,289)** |
| --- | --- | --- | --- |
| **Zip code** |  |  |  |
| 94110 | 3590 (100%) | 2484 (69.2%) | 1106 (30.8%) |
| 94112 | 1453 (100%) | 1242 (85.5%) | 211 (14.5%) |
| 94124 | 452 (100%) | 366 (81.0%) | 86 (19.0%) |
| 94102 | 344 (100%) | 274 (79.7%) | 70 (20.3%) |
| 94134 | 492 (100%) | 407 (82.7%) | 85 (17.3%) |
| 94103 | 499 (100%) | 356 (71.3%) | 143 (28.7%) |
| Other San Francisco/Bay Area | 4268 (100%) | 2680 (62.8%) | 1588 (37.2%) |

**Note:** proportions represent row percentages
