# Supplementary material for: A multi-component, community-based strategy to facilitate COVID-19 vaccine uptake among Latinx populations: From theory to practice
Source: PLoS One. 2021 Sep 20;16(9):e0257111. doi: 10.1371/journal.pone.0257111 (PMC8452046; doi:10.1371/journal.pone.0257111)
Supplement: S3 Table — (DOCX) [file pone.0257111.s004.docx]

**S3 Table. Characteristics of individuals who were offered participation in the on-site post-vaccination survey, according to whether they did or did not agree to complete the survey.**

**Note:** All surveys were completed between May 2 and May 19^th^, 2021.

|  | **Offered survey**  **(n=3,597)** | **Completed survey**  **(n=997)** | **Declined survey**  **(n=2,540)** |
| --- | --- | --- | --- |
| **Median age, IQR** | 37 (27-48) | 35 (26-46) | 38 (28-49) |
| **Age Category** |  |  |  |
| 16-30 | 1154 (32.6%) | 384 (38.5%) | 770 (30.3%) |
| 31-50 | 1676 (47.4%) | 430 (43.1%) | 1246 (49.1%) |
| 50-64 | 580 (16.4%) | 163 (16.3%) | 417 (16.4%) |
| 65 and older | 127 (3.6%) | 20 (2.0%) | 107 (4.2%) |
| **Sex** |  |  |  |
| Male | 2130 (60.2%) | 575 (57.7%) | 1555 (61.2%) |
| Female | 1359 (38.4%) | 409 (41.0%) | 950 (37.4%) |
| Non-binary/other | 48 (1.36%) | 13 (1.3%) | 35 (1.38%) |
| **Ethnicity** |  |  |  |
| Latinx | 2632 (74.4%) | 669 (67.1%) | 1963 (77.3%) |
| White | 427 (12.1%) | 163 (16.3%) | 264 (10.4%) |
| Asian | 242 (6.8%) | 83 (8.3%) | 159 (6.3%) |
| Black | 80 (2.3%) | 25 (2.5%) | 55 (2.2%) |
| Other | 156 (4.4%) | 57 (5.7%) | 99 (3.9%) |
