# Supplementary material for: A multi-component, community-based strategy to facilitate COVID-19 vaccine uptake among Latinx populations: From theory to practice
Source: PLoS One. 2021 Sep 20;16(9):e0257111. doi: 10.1371/journal.pone.0257111 (PMC8452046; doi:10.1371/journal.pone.0257111)
Supplement: S4 Table — (DOCX) [file pone.0257111.s005.docx]

**S4 Table. Acceptability measures associated with the Unidos en Salud vaccination site.**

|  | **Overall**  **(n=997)** | **Latinx**  **(n=669)** | **Not Latinx**  **(n=328)** | **P-value** |
| --- | --- | --- | --- | --- |
| **Clients stating they would recommend the UeS vaccination site to others** |  |  |  |  |
| Yes | 954 (98.6%) | 643 (98.2%) | 311 (99.4%) | 0.25 |
| No | 14 (1.5%) | 12 (1.8%) | 2 (0.6%) |  |
| **Who would you recommend this site to?** |  |  |  |  |
| Family members | 782 (82.1%) | 524 (81.6%) | 258 (83.0%) | 0.970 |
| Friends | 805 (84.5%) | 511 (79.6%) | 294 (94.5%) | <0.001 |
| Co-workers | 639 (67.1%) | 400 (62.3%) | 239 (76.8%) | <0.001 |
| **Features of the UeS vaccination that clients liked the most (top choice)** |  |  |  |  |
| Fast and efficient | 284 (32.8%) | 161 (27.5%) | 123 (44.1%) | <0.001 |
| Friendly and professional staff | 349 (40.3%) | 268 (45.7%) | 81 (29.0%) |  |
| Bilingual staff | 62 (7.2%) | 58 (9.9%) | 4 (1.4%) |  |
| Staff were available and able to answer questions | 14 (1.6%) | 11 (1.9%) | 3 (1.1%) |  |
| Getting vaccinated in their community | 77 (8.9%) | 42 (7.2%) | 35 (12.5%) |  |
| Getting vaccinated outside of a formal healthcare setting | 13 (1.5%) | 6 (1.0%) | 7 (2.5%) |  |
| Ease of booking 2^nd^ appointment | 32 (3.7%) | 16 (2.7%) | 16 (5.7%) |  |
| No requirement to show documentation | 30 (3.5%) | 20 (3.4%) | 10 (3.6%) |  |
| Other | 4 (0.5%) | 4 (0.7%) | 0 |  |
| **Features of the UeS vaccination that clients liked the most (choose all)** |  |  |  |  |
| Fast and efficient | 694 (69.6%) | 421 (62.9%) | 273 (83.2%) | <0.001 |
| Friendly and professional staff | 854 (85.7%) | 558 (83.4%) | 296 (90.2%) | 0.005 |
| Bilingual staff | 497 (49.8%) | 368 (55.0%) | 129 (39.3%) | <0.001 |
| Staff were available and able to answer questions | 373 (37.4%) | 226 (33.8%) | 147 (44.8%) | <0.001 |
| Getting vaccinated in their community | 428 (42.9%) | 252 (37.7%) | 176 (53.7%) | <0.001 |
| Getting vaccinated outside of a formal healthcare setting | 259 (26%) | 141 (21.1%) | 118 (36.0%) | <0.001 |
| Ease of booking 2^nd^ appointment | 492 (49.3%) | 273 (40.8%) | 219 (66.8%) | <0.001 |
| No requirement to show documentation of residency or vaccine eligibility | 276 (27.7%) | 176 (26.3%) | 100 (30.5%) | 0.19 |
| Other | 6 (0.6%) | 3 (0.4%) | 3 (0.9%) | 0.40 |

**Note:** All data is drawn from a survey among vaccinated clients aged ≥16 years old conducted after their first or second vaccine dose between May 2 and 19^th^, 2021 (n=997).
