# Supplementary material for: A multi-component, community-based strategy to facilitate COVID-19 vaccine uptake among Latinx populations: From theory to practice
Source: PLoS One. 2021 Sep 20;16(9):e0257111. doi: 10.1371/journal.pone.0257111 (PMC8452046; doi:10.1371/journal.pone.0257111)
Supplement: S5 Table — (DOCX) [file pone.0257111.s006.docx]

**S5 Table. Characteristics of clients receiving at least one vaccine dose at the Unidos en Salud vaccination site between February 1 and April 21, 2021, according to whether they completed both vaccine doses.**

|  | **Total clients receiving at least one dose**  **(n=9,305)** | **Completed both doses**  **(n=9,152)** | **Did not complete both doses**  **(n=153)** |
| --- | --- | --- | --- |
|  |  |  |  |
| **Age Category** |  |  |  |
| 16-30 | 1900 (100%) | 1865 (98.2%) | 35 (1.8%) |
| 31-50 | 3822 (100%) | 3752 (98.2%) | 70 (1.8%) |
| 50-64 | 2353 (100%) | 2321 (98.6%) | 32 (1.4%) |
| 65 and older | 1230 (100%) | 1214 (98.8%) | 16 (1.2%) |
| **Sex** |  |  |  |
| Male | 4272 (100%) | 4221 (98.8%) | 51 (1.2%) |
| Female | 4851 (100%) | 4755 (97.8%) | 96 (2.2%) |
| Non-binary/other | 182 (100%) | 176 (96.7%) | 6 (3.3%) |
| **Ethnicity** |  |  |  |
| Latinx | 6313 (100%) | 6207 (98.3%) | 106 (1.7%) |
| White | 1476 (100%) | 1453 (98.4%) | 23 (1.6%) |
| Asian | 760 (100%) | 751 (98.8%) | 9 (1.2%) |
| Black | 230 (100%) | 227 (98.7%) | 3 (1.3%) |
| Other | 526 (100%) | 514 (97.7%) | 12 (2.3%) |

**Note:** Analysis is censored after April 21, 2021 in order to allow all clients equal opportunity to accrue 4 weeks of follow-up time after their first vaccine dose at the neighborhood vaccination site. There were 1,780 people excluded who received their 1^st^ dose after April 21, 2021 and 13 excluded who received only their second dose at the neighborhood site.
