# Supplementary material for: A multi-component, community-based strategy to facilitate COVID-19 vaccine uptake among Latinx populations: From theory to practice
Source: PLoS One. 2021 Sep 20;16(9):e0257111. doi: 10.1371/journal.pone.0257111 (PMC8452046; doi:10.1371/journal.pone.0257111)
Supplement: S1 Appendix — (PDF) [file pone.0257111.s007.pdf]

S1 Appendix. Post-vaccination survey

Thank you for choosing our vaccination site at 24th & Capp – we enjoyed seeing you. We would like to learn more about your experience at our vaccine site. Your honest responses to the questions below will help us make improvements to our vaccine operations and help us advocate for more effective vaccine outreach. All information will be kept confidential. Thank you!

Gracias por elegir nuestro sitio de vacunación en la 24th & Capp - fue un gusto servirle. Sin embargo nos gustaría saber más sobre su experiencia en nuestro sitio de vacunación. Con su colaboración en responder a las siguientes preguntas nos ayudará a mejorar nuestro servicio de vacunación, esto nos permitirá poder difundir el programa de vacunación que tenemos en esta área de la comunidad. Siéntase seguro que la información que usted nos proporcione es totalmente confidencial. ¡Gracias!

Language

◯ English
◯ Español

____

____

First Name __________________________________

Last Name __________________________________

Phone Number __________________________________

Demographics

What is your current primary occupation?

◯ Food/beverage
◯ Healthcare
◯ Tradesperson (construction, plumbing), cleaning (janitor, housekeeper), or personal services (hairdresser)
◯ Education
◯ Finance, sales, and technology
◯ Student
◯ Retired/homemaker
◯ Unemployed
◯ Other

Other Occupation

____

What is your yearly household income?

◯ Less than $50,000 per year
◯ Between $50,000-100,000 per year
◯ More than $100,000 per year

Are you a first or second generation immigrant? Note: We are asking this question to better understand attitudes toward vaccine uptake and access in immigrant communities. This data will be kept strictly confidential and will not be reported.

◯ Not a first or second generation immigrant
◯ First-generation immigrant (born outside the country)
◯ Second-generation immigrant (at least one parent born outside the country)

Do you have health insurance?

◯ Yes
◯ No

If so, what type of insurance do you have?

◯ Private insurance (Kaiser, Anthem, Blue Cross)
◯ Public insurance (Medi-Cal, Medicare, Healthy San Francisco)
◯ I don't know

Do you have a personal doctor (primary care provider)?

◯ Yes
◯ No

Choosing Our Vaccine Site

How did you find out about our vaccine site?

◯ I received an invitation on my phone
◯ I was referred from the testing site
◯ An outreach volunteer came to my workplace/home and told me about it
◯ I passed by the site
◯ I saw a flyer for the site
◯ I heard about it from a friend, family member, or co-worker
◯ I saw it on social media (Facebook, Instagram, Tik-Tok)
◯ I saw it in the news (newspaper, TV, radio)
◯ Other

Other

____

Why did you choose to get vaccinated at our vaccine site? Select all that apply.

☐ I was not aware of other options
☐ It was in my neighborhood
☐ Staff are bilingual
☐ Scheduling was easy, efficient, and convenient
☐ I had a previous positive experience at the testing site
☐ I had a positive interaction with the Unidos en Salud or Latino Task Force staff/volunteers
☐ Someone I trusted invited me or suggested it to me
☐ I tried to get vaccinated somewhere else and could not
☐ Other

Other

____

What is the MAIN REASON you chose to get vaccinated at our site? Please select one option.

◯ I was not aware of other options
◯ It was in my neighborhood
◯ Staff are bilingual
◯ Scheduling was easy, efficient, and convenient
◯ I had a previous positive experience at the testing site
◯ I had a positive interaction with the Unidos en Salud or Latino Task Force staff/volunteers
◯ Someone I trusted invited me or suggested it to me
◯ I tried to get vaccinated somewhere else and could not
◯ Other

Other

____

If this site had not existed, do you think you would have been vaccinated earlier, later, or about the same time?

◯ Earlier
◯ About the same time
◯ Later

Experience at Our Vaccine Site

What did you like about our vaccine site? Select all that apply.

☐ Staff are friendly and professional
☐ Staff are bilingual
☐ It was fast and efficient
☐ I liked being vaccinated in my community
☐ I liked being vaccinated outside a formal healthcare setting
☐ There are staff available to answer my questions
☐ I was able to book my 2nd appointment easily
☐ I did not have to provide documentation for my residency, occupation status, or employment
☐ Other

Other

____

What did you LIKE MOST about our vaccine site? Please select one option.

◯ Staff are friendly and professional
◯ Staff are bilingual
◯ It was fast and efficient
◯ I liked being vaccinated in my community
◯ I liked being vaccinated outside a formal healthcare setting
◯ There are staff available to answer my questions
◯ I was able to book my 2nd appointment easily
◯ I did not have to provide documentation for my residency, occupation status, or employment
◯ Other

What did you not like about the vaccine site? Select all that apply.

☐ It was too public
☐ The wait time was too long
☐ I did not like the setting/environment
☐ I did not have positive interactions with staff
☐ My questions about COVID-19 and the vaccine were not answered
☐ I had difficulty with my 2nd dose scheduling
☐ Other
☐ None of the above

Did you dislike anything our vaccination site?

◯ Yes
◯ No

Please specify

Do you have suggestions or comments for how we can improve our vaccination site?

◯ Yes
◯ No

Other suggestions or comments

Vaccinations Among Your Contacts

Would you recommend this vaccination site?

◯ Yes
◯ No

Why or why not?

If so, who would you recommend us to? Select all that apply.

☐ Family Members
☐ Friends
☐ Co-workers

Do you have any family members, friends, or coworkers who live in the Bay Area (age 16 or older) who have not been vaccinated?

◯ Yes
◯ No

If yes, how many people do you know who live in the Bay Area and have not been vaccinated?

◯ 1-2 people
◯ 3-5 people
◯ 6-10 people
◯ More than 10 people

If yes, who? Select all that apply.

☐ Friends
☐ Family members
☐ Co-workers

If yes, do you have suggestions for what we could do to help reach them for vaccination?

◯ Yes
◯ No

Suggestions for reaching those unvaccinated

Since being vaccinated, are you more likely to recommend getting vaccinated to any friends, family members or colleagues because of your experiences at this site?

◯ Yes
◯ No

If yes, approximately how many friends, family members or colleagues have you had (or will you have) these discussions with to recommend that they vaccinated?

◯ 0 people
◯ 1-2 people
◯ 3-5 people
◯ 6-10 people
◯ More than 10 people

How many friends, family members or colleagues are you aware of that got vaccinated after you shared your experiences and recommended that they get vaccinated?

◯ 0 people
◯ 1-2 people
◯ 3-5 people
◯ 6-10 people
◯ More than 10 people
